# Inequalities in Unmet Oral Health Care Need Among Adults in East Iran, a Cross Sectional Population-Based Study

**DOI:** 10.30476/DENTJODS.2020.86319.1188

**Published:** 2021-09

**Authors:** Zahra Yaghoubi, Tayebeh Malek Mohammadi, Mohammad Khajedaluee

**Affiliations:** 1 Dept. of Community Oral Health, School of Dentistry, Mashhad University of Medical Sciences, Mashhad, Iran; 2 Social Determinants of Health Research Center, Institute for Futures Studies in Health, Dept. of Dental Public Health, Kerman, Iran; 3 Dept. of Community Medicine, School of Medicine, Mashhad University of Medical Sciences, Mashhad, Iran

**Keywords:** Oral health, Needs and demand, Socioeconomic status, Adults

## Abstract

**Statement of the Problem:**

Need assessment is considered as a key element of health care planning. Subjective measures can be useful tools in epidemiologic surveillances.

**Purpose:**

The aim of study was to evaluate inequality in perceived unmet oral health need (PUOHN) in adults in east of Iran.

**Materials and Method:**

In this cross-sectional population-based study, the target population included adult residents in the Mashhad and Kerman city. Data was collected through telephone interviews using a validated structured questionnaire. Phone numbers were obtained from telecommunication company. Participants were selected by stratified random sampling. Predisposing and enabling variables associated with PUOHN were included gender, age, educational level, job, insurance coverage, dental insurance, type of insurance, residential location, household size, and family economic indicators. Logistic regression was used to examine association of PUOHN and predisposing and enabling factors.

**Results:**

1475 individuals participated in the study (response rate of 63%). 52% of participants stated that their dental needs have not been met during the past year. The mean ages of respondents were 39 years old and 69.8% were female. Logistic regression analyses indicated living in rental house [OR=2 (95% CI 1.25-3.21), *p* Value=0.004] and higher household size [OR=1.19(95% CI 1.003-1.43), *p*= 0.04] significantly associated with PUOHN.

**Conclusion:**

The results of this study indicated high PUOHN in the adult population of East Iran. Effective strategies must be implemented to provide accessible dental services regardless of socioeconomic status.

## Introduction

Oral health is one of the key elements of overall health [ 1 ]. Oral diseases are known as one of important public health issues due to their adverse effect on general health and quality of life, and high cost of treatment [ 1 ]. Oral health need assessment considered as a key element of oral health care planning [ 2 ]. The advantages of community health need assessment can be summarized as measuring the burden of diseases, determining differences in patterns of need in populations, identifying patient’s priorities, realistic estimate of needed health care interventions, and cost-effective allocation of limited resources [ 2 ]. Bradshaw [ 3 ] has described three types of need, which can be applied in the health context; normative need is determined by clinician and professional staffs, perceived (felt) need is refection of self-assessment of health need, and expressed need is a perceived need that has led to receipt of services. Although the most commonly used method of assessing need for dental care is solely based on clinical criteria, reliance on merely clinical indicators suffers from several limitations including deficiency in objectivity and reliability, neglect of health behaviors and patient compliance, disregard of patient’s attitude and consumer rights, and neglect of quality of life concept [ 2 , 4 ]. Gift *et al*. [ 5 ] presented a model showing subjective perception of oral health was affected by demographic, enabling and predisposing factors. Financial capabilities such as income, insurance and level of education are considered as enabling factors. It should be taken into account that enabling factors do not predict demanding services since the key determinative factor for receiving services is perception of the need [ 6 ].

The role of socioeconomic factors as important determinants of oral health in different communities has increasingly been noticed. According to the World Health Organization (WHO) report, inequality in oral health care is a major research priority in the 21^st^ century [ 7 ]. The issue was clearly evidenced by studies conducted in developed countries so that socioeconomic inequalities have been observed in subjective oral health [ 8 - 9 ]. However, due to the lack of sufficient research in developing countries, more investigations in these areas are needed. Besides, few studies regarding subjective oral health and socioeconomic inequalities in adults have conducted in Iran. In Ghorbani *et al*.’s study [ 10 ] in Tehran, despite the significant association of number of self-reported non-replaced extracted teeth and lower level of education and wealth index, there was no similar association of perceived oral health with socioeconomic levels. During the last three decades, Iran Ministry of Health and Education has tried to provide basic dental services by prioritizing deprived groups in underserved areas like rural and suburban regions. Little is known about the impact of this strategy on the extent of subjective oral health inequality. In literature review, we could not find any recently published study in this regard performed outside of Iran’s capital. To clarify the issue, the aim of this study was to measure perceived unmet oral health need (PUOHN) across different socioeconomic indicators in Mashhad and Kerman, two of the largest cities in east of Iran.

## Materials and Method

### Design and study population

The aim of this cross-sectional study was to assess inequalities in PUOHN in adult population (18 and over) of Mashhad and Kerman cities by telephone interview. All the phone numbers were obtained from Telecommunication Company. Phone numbers were categorized into 35 urban regions (28 regions in Mashhad, 7 regions in Kerman). 34 percent of regions were located in suburban areas (Mashhad 10, Kerman 2). To reach a representative sample, estimated sample size was distributed proportionally among urban regions based on stratified random sampling. At first, according to population of the two cities, the sample size was distributed (Mashhad 1100, Kerman 375). In the next step, exact required sample size of each urban region was calculated based on proportion of the number of telephone lines in each urban region to the entire city phone lines. To compensate for non-respondent or busy phone lines, five-fold numbers of determined sample size, random phone numbers were obtained by Excel “RAND BETWEEN” function. Kerman University of Medical Science granted ethical approval for the study [number IR.kmu.REC.1394. 549]. Correspondingly, informed consent was obtained from the participants at the beginning of the interview.

### Interview

We performed a systematic review on questionnaires on PUOHN. The result implied that comprehensive questionnaires were not available [ 11 ]. Therefore, a structured questionnaire was designed. Firstly, the developed questionnaire was revised based on qualitative validation process. In the next step, content validity index and content validity ratio were assessed. Except for two deleted items, all of the questions gained the acceptable scores. Reliability was assessed by test-retest method and use of Cronbach’s alpha. The results of reliability assessment were acceptable too. The development and validation process of the questionnaire has also been reported [ 12 ]. The telephone interview was conducted by three trained interviewers. Before performing the main part of the project and during the pilot study, the interview process was reviewed and some corrections were made in the questionnaire. Each interview lasted about seven minutes. Non-respondent or in-use lines were withdrew from the call list after two attempts. Phone calls were made in the morning and evening of workdays from June to October 2016.

### Study variables

#### Outcome

The outcome, PUOHN was evaluated by the following question “during the past year, do you think your dental needs were met? Yes, no”

#### Independent variables

Predisposing variables potentially associated with PUO-HN included gender, age and educational level (illiterate, elementary school, middle school, high school or diploma, associate degree, bachelor’s degree and over). Enabling variables were job (employee, self-employed, worker, housewife, student, unemployed), insurance coverage, dental insurance, type of insurance (private or public insurance), residential location (urban or sub urban area according to the phone number region), household size, and family socioeconomic indictors such as the use of personal car or public transport, monthly family income (Iranian Rial), house area (m^2^), and house ownership (own, rent).

### Statistical analysis

Statistical analysis was performed using SPSS software (version 20). Bivariate analyses were conducted to determine significant predisposing and enabling variables associated with PUOHN. Then, significant predictor variables were fitted in logistic regression model. 

## Results

To reach 1475 interview, 7291 phone calls were made. Of all 2854 answered calls, 630 were commercial lines, 1475 individuals participated in the study and 749 were not willing to participate (response rate of 63%). Characteristics of the studied population are presented in Table 1. 

**Table 1:** Descriptive and bivariate analysis results for PUOHN according to demographic of studied population binary analysis of socio-demographic characteristics of participants by PUOHN

Variables	Mean or (%)	PUOHN (Perceived Unmet Oral Health Need)(%)	*p* value (chi-square#)
Gender			0.97
Male	30.2	50.1	
Female	69.8	50.2	
Age			0.228
18-30	29.4	47.5	
31-45	41.7	53.5	
46-60	18.4	50.4	
>60	10.5	46.2	
Education level			0.012
Illiterate	3.7	42	
Elementary school	12	50.9	
Middle school	10.4	60	
High school or diploma	38.1	54.1	
Associates degree	7.7	51	
Bachelor’s degree and over	28.1	44.4	
Job			<0.001
Employee	17.6	40.3	
Worker	1.5	75	
Self-employed	22.2	56.2	
Student	4.3	28.1	
House wife	52.9	51.6	
Unemployed	1.5	75	
Insurance			0.079
Yes	89.1	49.4	
No	10.9	57	
Dental insurance			< 0.001
Yes	33.7	38.1	
No	47.8	53.3	
Unknown	18.5	43.5	
Residential area			0.897
Urban	75.4	50.3	
Sub urban	24.6	50.7	
House ownership			0.001
Owned	62.7	53.8	
Rental	36.1	65.5	
Other	1.2	81.1	
Transportation			< 0.001
Personal car	56.9	51.9	
Public transport	39.6	63.3	
Other	3.5	70.4	
Type of insurance			0.113
Public (governmental)	78.3	51.9	
Private(supplemental)	18.5	44	
Unknown	3.2	47.2	
Income (Rial)	16900000	900000-150000000*	0.022#
Family members	3.8	1-11*	0.029#
House area(square meter)	117	20-1000*	0.007 #

* For continuous variables, range (minimum; maximum) is presented.

# association of continuous variables with binary PUOHN was tested by using logistic regression.

Respondents were mostly female (69.8%) and aged between 30 to 45 years [mean age 39 (standard deviat-ion 13.73) years old]. More than half of respondents (52%) stated that during past year, their dental need has not been met. Bivariate analysis was used to test the relationship between PUOHN and predisposing and enabling factors including demographic and socioeconomic variables. Insurance coverage, type of insurance (public, private), residential area (urban, suburban) and age were not statistically significant. Then, the other significant variables including income, dental insurance, job, status of house ownership (rental, own-ed), family members, and type of transport (personal car, public transport) were fitted in the logistic regression analysis. The results showed living in rental house [OR=2, (95% CI 1.25-3.21), *p*= 0.004] and higher hou-sehold size [OR=1.19 (95% CI 1.003-1.43), *p*= 0.04] were significantly associated with PUOHN. The resul-ts of logistic regression analysis are presented in Table 2. 

**Table 2:** Logistic regression results for PUOHN (Perceived Unmet Oral Health Need) during the past year

Variables	*p* Value	OR (95% CI)
House area	0.65	0.999(0.996-1.003)
Income	0.09	1.00(1.00-1.00)
Education	0.38	1.089(0.9-1.32)
Dental insurance	0.14	1.29(0.91-1.83)
Family members	0.04	1.19(1.003-1.43)
transportation	0.07	1.07(0.99-1.17)
House ownership	0.004	2.009(1.25-3.21)
Job	0.44	1.06(0.91-1.24)

Significant *p* Values are bold

## Discussion

This is the first study to assess socioeconomic gradient in PUOHN in representative sample of east of Iran. The results indicated more than half of Mashhad and Kerman residents perceived their dental needs have not been met. Internationally, studies imply wide range of PUOHN. Although more studies show the prevalence of PUOHN below 50% [ 13 - 16 ], the results similar to our study have been reported in some developed [ 17 - 18 ] as well as developing countries [ 19 - 21 ]. Higher PUOHN has been reported in few studies [ 22 - 23 ]. In addition, far lesser PUOHN (10% or lower) have been reported in other studies in both developed and developing countries [ 24 - 28 ]. Explaining observed variation in PUOHN in different communities is a complex issue; differences in oral health care system, insurance coverage, target population, culture and the methods used for investigating PUOHN should be considered. Hence, reported PU-OHN varies hugely in different studies in a country across adults target populations like racial and ethnic minorities, refugees, disabled community dwelling adults.

In Iranian health care system, private sector is known to be the main and most accessible provider of dental care [ 10 ]. Public sector delivers limited basic dental services like oral examination, extraction, and preventive services. Clearly, delivering dental services in private sector is more costly and lack of dental insurance is a probable barrier to utilize dental care. 

By using the logistic regression model, predisposing factors including gender, age and educational level did not show significant statistical association with PUOHN. Association between gender and PUOHN is equivocal; the result of this study is consistent with the finding of studies in United States [ 17 ], Pakistan [ 19 ], Sri Lanka [ 16 ], and Indonesia [ 26 ]. While the studies in Tehran [ 29 ], Brazil [ 24 ], United States [ 30 ] and Tanzania [ 31 ] reported that women had more PUOHN, other studies imply on higher PUOHN among men [ 30 , 32 ]. In the present study, there was no association between age and PUOHN, which was in line with a study performed in Pakistan [ 19 ]. It should also be taken into account that other related studies have indicated higher PUOHN in the middle-aged adults [ 24 , 26 ] followed by a decrease in elderly [ 16 , 32 ]. This may be due to higher prevalence of complete tooth loss and use of dentures in old age.

In the current study, no association was found between PUOHN and the level of education, like other studies in Pakistan [ 19 ], Seri Lanka [ 16 ], and Tanzania [ 31 ]; however, other studies in Brazil [ 24 ], United States [ 13 ] and European countries [ 32 ] showed higher PUOHN in lower level of education. Insurance is considered an enabling factor for receiving dental services but in this study, it was not related to PUOHN, which might be due to the lack of insurance coverage for the routine dental services in Iran [ 33 ]. Contradictorily, Bayat *et al*.’s study [ 29 ] in Tehran showed association between demand for oral health care services and insurance coverage. Studies in United States [ 30 ], European countries [ 32 ] and Indonesia [ 26 ] also showed lower PUOHN among underinsured people. To evaluate the role of enabling factors in PUOHN inequality, we used a series of socioeconomic indicators. Employment did not associate with PUOHN; however, some studies showed statistical association between unemployment (losing job) and PUOHN [ 34 ]. Moreover, income did not have a significant effect on PUO-HN, which is similar to the results of some other developing countries such as Pakistan [ 19 ] and Sri Lanka [ 16 ]. Living in urban or sub urban areas did not show association with PUOHN. It may be due to public primary oral health care delivery in the sub urban regions.

The recognized significant economic variables were living in rental home and household’s size. The result agreed with findings of some studies in European countries. They showed living in rental house and larger size of household (particularly single parents), associate with PUOHN [ 32 ]. Socioeconomic gradient in PUOHN was found in Indonesia [ 26 ] and Spain [ 27 ] too. Evaluation of PUOHN in some European countries implied on determinative role of economic class in meeting dental health needs, so that PUOHN in the most deprived groups is three times more than the most prosperous economic class [ 32 ]. In Ghorbani et al.’s study [ 10 ] in Tehran, no association was found between perceived oral health and socioeconomic class while another study in Tehran concluded socioeconomic inequality affect accessing dental health care. It should be noticed that people of lower socioeconomic classes may have less complaint of oral problems; they try to tolerate oral problems for longer time; and therefore, PUOHN would be affected [ 10 ].

Despite the advantages of having a large representative sample size and studying a wide range of socioeconomic variables potentially related to PUOHN, there were some drawbacks. To obtain equal gender distribution, telephone interviews were conducted in the afternoon and early evening when the presence of employed men is more probable, ultimately the participation of women was higher. Although need to dental care was evaluated subjectively, some studies have referred to the usefulness of subjective methods in population need assessments and their association with clinical findings [ 22 , 35 ]. It seems that the validity of self-reporting economic information may be questionable. To improve the validity of self-reported economic items, we tried to use various economic indicators.

## Conclusion

Our findings showed high PUOHN in the adult population of East Iran. Apart from improving socioeconomic conditions as a decisive step, effective strategies must be implemented to provide accessible dental services regardless of socioeconomic status.
